# Atrial fibrillation as a new prognosis factor in chronic patients after hospitalization: the CHRONIBERIA index

**DOI:** 10.1038/s41598-023-30610-2

**Published:** 2023-03-11

**Authors:** Javier Suarez-Dono, Ignacio Novo-Veleiro, Francisco Gude-Sampedro, Ricardo Marinho, Sara Xavier-Pires, Diana Rocha, João Araújo-Correia, Cecília Moreira, Francisca Beires, Danay Pérez, Filipa David, J. Vasco-Barreto, Esther Del Corral-Beamonte, Juan-Carlos Piñeiro-Fernández, Emilio Casariego-Vales, Jesús Diez-Manglano, Antonio Pose-Reino

**Affiliations:** 1 Internal Medicine Department, Complexo Hospitalario Universitario de Santiago de Compostela y Barbanza, School of Medicine, University of Santiago de Compostela, Rua da Choupana S/N, 15706 Santiago de Compostela. A Coruña, Spain; 2 Epidemiology Unit, Complexo Hospitalario Universitario de Santiago de Compostela y Barbanza, School of Medicine, University of Santiago de Compostela, Santiago de Compostela, Spain; 3 Internal Medicine Department, Santo António Hospital - Centro Hospitalar e Universitário do Porto (CHUP), Porto, Portugal; 4 Multidisciplinary Biomedical Research Unit (UMIB), Abel Salazar Biomedical Science Institute (ICBAS), Porto, Portugal; 5 Internal Medicine Service, Hospital Pedro Hispano, Matosinhos Local Health Unit, Matosinhos, Portugal; 6 Abel Salazar Biomedical Science Institute (ICBAS), Porto, Portugal; 7 Internal Medicine Department. Hospital Royo Villanova, School of Medicine, University of Zaragoza, Zaragoza, Spain; 8 Internal Medicine Department, Hospital Lucus Augusti, Lugo, Spain

**Keywords:** Cardiovascular diseases, Geriatrics, Health services, Epidemiology

## Abstract

A collaborative project in different areas of Spain and Portugal was designed to find out the variables that influence the mortality after discharge and develop a prognostic model adapted to the current healthcare needs of chronic patients in an internal medicine ward. Inclusion criteria were being admitted to an Internal Medicine department and at least one chronic disease. Patients’ physical dependence was measured through Barthel index (BI). Pfeiffer test (PT) was used to establish cognitive status. We conducted logistic regression and Cox proportional hazard models to analyze the influence of those variables on one-year mortality. We also developed an external validation once decided the variables included in the index. We enrolled 1406 patients. Mean age was 79.5 (SD = 11.5) and females were 56.5%. After the follow-up period, 514 patients (36.6%) died. Five variables were identified as significantly associated with 1 year mortality: age, being male, lower BI punctuation, neoplasia and atrial fibrillation. A model with such variables was created to estimate one-year mortality risk, leading to the CHRONIBERIA. A ROC curve was made to determine the reliability of this index when applied to the global sample. An AUC of 0.72 (0.7–0.75) was obtained. The external validation of the index was successful and showed an AUC of 0.73 (0.67–0.79). Atrial fibrillation along with an advanced age, being male, low BI score, or an active neoplasia in chronic patients could be critical to identify high risk multiple chronic conditions patients. Together, these variables constitute the new CHRONIBERIA index.

## Introduction

Over the last decade, the relevance of chronicity has increased exponentially as there is a high percentage of people with chronic diseases, advanced age and multiple comorbidities, mainly in developed countries^1^. In Spain, the last European Health Survey conducted in 2014 reflected that 10.6% of all the adult population had at least one chronic disease, a percentage which dramatically increased with age^2^. In Portugal, with a higher percentage of elderly population, the situation is similar.

Chronic patients account for up to 70% of total admissions to Internal Medicine departments and, during their hospitalization period, they can suffer multiple complications related to comorbidity, frailty or age-related conditions^3^. For this reason, and also because of the huge healthcare resources that these patients usually use, making an appropriate classification and correctly identifying those in high risk of complications and mortality is most essential. Over the last decades, a great number of scores have been developed—most of them focused on one single disease—to predict poorer outcomes in chronic patients^4–6^. There are also general scores and indexes aimed at the evaluation of chronic patients, like the Chronic Disease Score (CDS)^7^, the Charlson Index or its updated version^8,9^, the Cumulative Index Rating Scale (CIRS), the Index of Coexisting Disease (ICED) or the Kaplan Index^10^. All these indexes and scores have some weaknesses that limit their generalized use in chronic patients, such as not including some relevant diseases in chronic patients, like anemia or chronic kidney disease (CKD) or the excessive importance given to other diseases, like the acquired immune deficiency syndrome (AIDS). Undoubtedly, this is due to the fact that at that time, in some cases more than 30 years ago, some specific chronic diseases predominated, which may or may not coincide with the present moment and whose prognostic weight has changed over the years, which raises the need to search for more adapted prediction models.

From a local perspective, the Spanish Internal Medicine Society developed in 2010 the PROFUND index, which was validated to accurately predict high mortality risk in patients with at least 2 chronic diseases affecting different organs or systems^11^. However, and despite its usefulness, it cannot be used as a proper classification method in all chronic patients. Moreover, other authors have developed scores specifically designed for older adults, regardless of comorbidity^12^ but again young chronic patients are ignored, which also limits their application to all chronic patients.

In order to use healthcare resources more appropriately and efficiently, the identification of high-risk chronic patients and a global stratification of this population in each territory play a capital role in the present and future of healthcare organization. In this sense, it would be of great interest if physicians could establish a correct mortality risk for every chronic patient when demanding assistance in order to achieve a better individualized planning, not only regarding clinical management, but also from a social and economic point of view^13^.

The Spanish government has addressed the reorientation of the healthcare of chronic patients by focusing on 3 key points: the identification of specific patient´s needs, the optimization of the pharmacological management of each chronic disease through the adoption of a comprehensive approach and ensuring effective, secure, efficient and sustainable healthcare based on the best available scientific evidence^14^. In light of these criteria, we believe that having useful tools to correctly stratify chronic patients could be one of the keys to achieve these objectives and, consequently, we developed and validated the CRONIGAL index in 2016, which calculates the one-year mortality risk of chronic patients after admission with seven variables^15^.

As the Spanish and Portuguese populations have similar characteristics, a collaborative project was designed in order to find out the risk factors that influence the mortality after discharge and based on variables easy to obtain from the clinical history, develop a simplified prognostic index, more generalizable, adapting it to the current healthcare needs of chronic patients in an internal medicine ward. We also wanted to confirm that the value of this index is maintained over time.

## Patients and methods

For the development of the present work, we used data from two prospective previous studies, one of them developed in the Healthcare Area of Santiago de Compostela in Galicia (North-Western Spain)^15^ and the other one in Aragon (North-Eastern Spain)^16^ and also added new data from two similar cohorts recruited in two hospitals in Portugal (Pedro Hispano Hospital in Matosinhos and Santo Antonio Hospital in Porto). Thus, the recruitment period took place in different phases at each center (Santiago de Compostela: June 2011–July 2012; Aragon: March–June 2011; Matosinhos and Porto: January–December 2019). Inclusion criteria were: previous history of at least one chronic disease which compromises the function of at least one organ, being admitted to the hospital in an Internal Medicine department and having completed the one-year follow-up through electronic medical history records or telephone interview. All patients were over 18 years old and all of them were recruited during a hospital admission for decompensation of their chronic disease, generally associated with several comorbidities. An information sheet was provided to all patients, and they all signed an informed consent form to participate in the present study. Patients who died during admission where excluded and also those who were lost to follow-up in the 1-year period after discharge. The study protocol was reviewed a and approved by the Ethics Committee of Clinical Investigations of Galicia (Spain) which certified that the present study was performed in accordance with all required guidelines and regulations.

Basic clinical and biochemical variables were coded, and all patients were evaluated on the basis of the Barthel index (BI) to measure their physical dependence. The Pfeiffer´s test (PT) was used to establish and grade the cognitive status of all enrolled cases^17,18^. We categorized patients according to the number of errors in Pfeiffer´s test as follows: grade 1 (0–2 errors), grade 2 (3–4 errors), grade 3 (5–7 errors), grade 4 (8 or more errors), grade 5 (patient is unable to answer due to severe cognitive impairment). We specifically evaluated those variables which our group previously reported as useful to predict mortality during the year after hospital discharge^15^.

An inferential and descriptive initial analysis was performed. Subsequently, we conducted logistic regression and Cox proportional hazard models to analyze the influence of different factors on one-year mortality. ROC curves were built to evaluate the ability of each model to discriminate through area under the curve (AUC) comparison. The Hosmer–Lemeshow and Brier tests were used to calibrate the different models.

Finally, an external validation was carried on, a few years later, selecting 250 patients with similar clinical characteristics of those included in the main study (Table 1). We compared the predicted results after discharge with the real evolution during 1-year follow up to assess this validation.

**Table 1:** Comparison among group of patients on the basis of enrollment site.

Variables	Group 1 (n = 245)	Group 2 (n = 249)	Group 3 (n = 443)	Group 4 (n = 469)	Validation cohort (n = 250)	*P*
Age	72.3 (13.7)	74.3 (13.7)	80.8 (8.9)	84.8 (7.3)	86 (6.8)	< 0.001
Being male	122 (49.8)	133 (53.4)	198 (44.7)	159 (33.9)	134 (53.6)	< 0.001
Neoplasia	65 (26.5)	41 (16.5)	53 (12)	26 (5.5)	28 (11.2)	< 0.001
BI punctuation	71.7 (32.5)	74.3 (27.8)	54.6 (35.4)	45.9 (31.8)	72.5 (26.2)	< 0.001
PT category						
1	147 (60)	151 (60.6)	166 (37.5)	151 (32.2)	–	< 0.001
2	37 (15.1)	42 (16.9)	80 (18.1)	58 (12.4)		
3	25 (10.2)	33 (13.3)	87 (19.6)	38 (8.1)		
4	15 (6.1)	10 (4)	54 (12.2)	21 (4.5)		
5	21 (8.6)	13 (5.2)	56 (12.6)	201 (42.9)		
Delirium	23 (9.4)	50 (20.1)	86 (19.8)	59 (12.6)	38 (15.2)	< 0.001
AF	52 (21.2)	86 (34.5)	187 (42.4)	157 (33.5)	121 (48.4)	< 0.001

Matosinhos (Group 1), Porto (Group 2), Aragon (Group 3) and Santiago de Compostela (Group 4).

BI: Barthel index, PT: Pfeiffer´s test, AF: Atrial fibrillation.

### Ethical approval

The study protocol was reviewed and approved by the Ethics Committee of Clinical Investigations of Galicia.

## Results

We enrolled 1406 patients with a mean age of 79.5 (SD = 11.5) years with a higher percentage of females (56.5%). Regarding the origin of patients, 245 (17.4%) were enrolled in Matosinhos (Group 1), 249 (17.7%) in Porto (Group 2), 443 (31.5%) in Aragon (Group 3) and 469 (33.4%) in Santiago de Compostela (Group 4). The characteristics of the patients enrolled in each group are shown in Table 1.

The median score in the Barthel index in the whole series was 60 [Inter-quartile Range (IQR) = 65] points and the distribution of Pfeiffer´s test categories as we previously defined them was: 615 patients (43.7%) in grade 1, 215 (15.4%) in grade 2, 183 patients (13%) in grade 3, 100 patients (7.1%) in grade 4 and 291 patients (20.7%) in grade 5. Other remarkable findings were that 185 patients (13.2%) had a diagnosis of an active neoplasia and 218 (15.6%) suffered an episode of delirium during hospital admission. 482 patients (34,3%) were diagnosed with atrial fibrillation.

**Figure 1:**
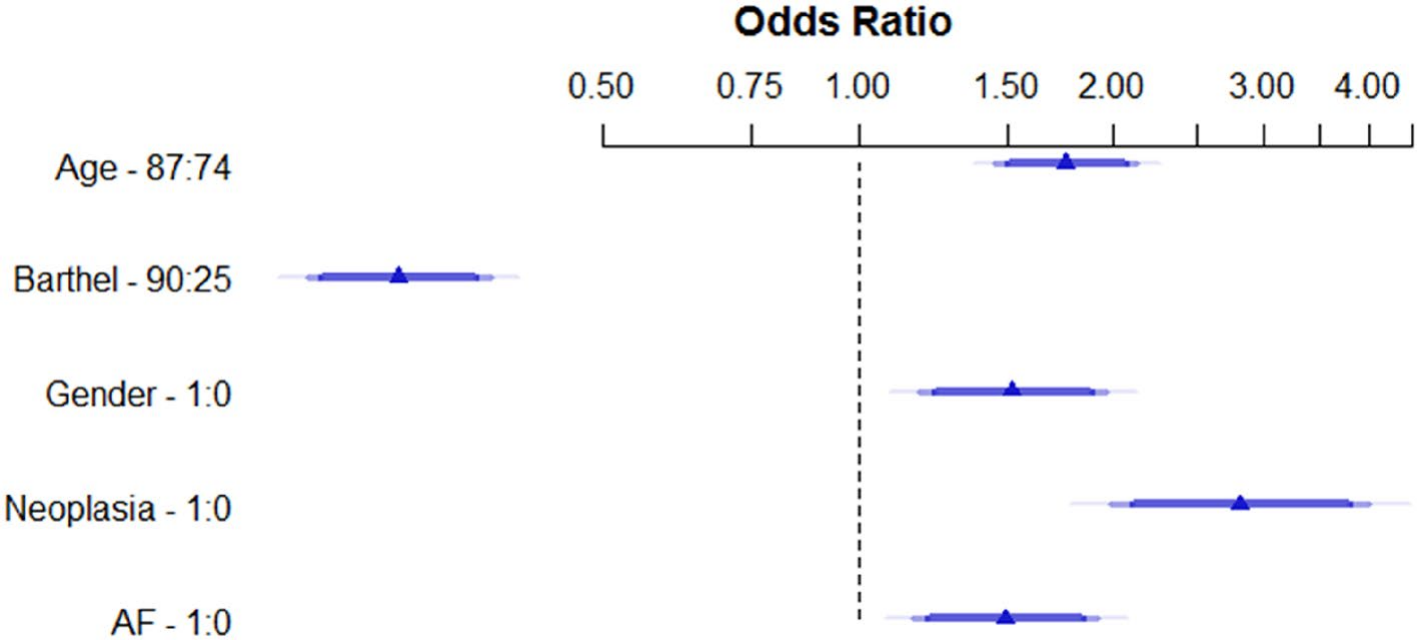
Multivariate analysis. Logistic regression analysis with the 5 variables identified as significantly associated to one-year mortality applied to the global sample.

**Figure 2:**
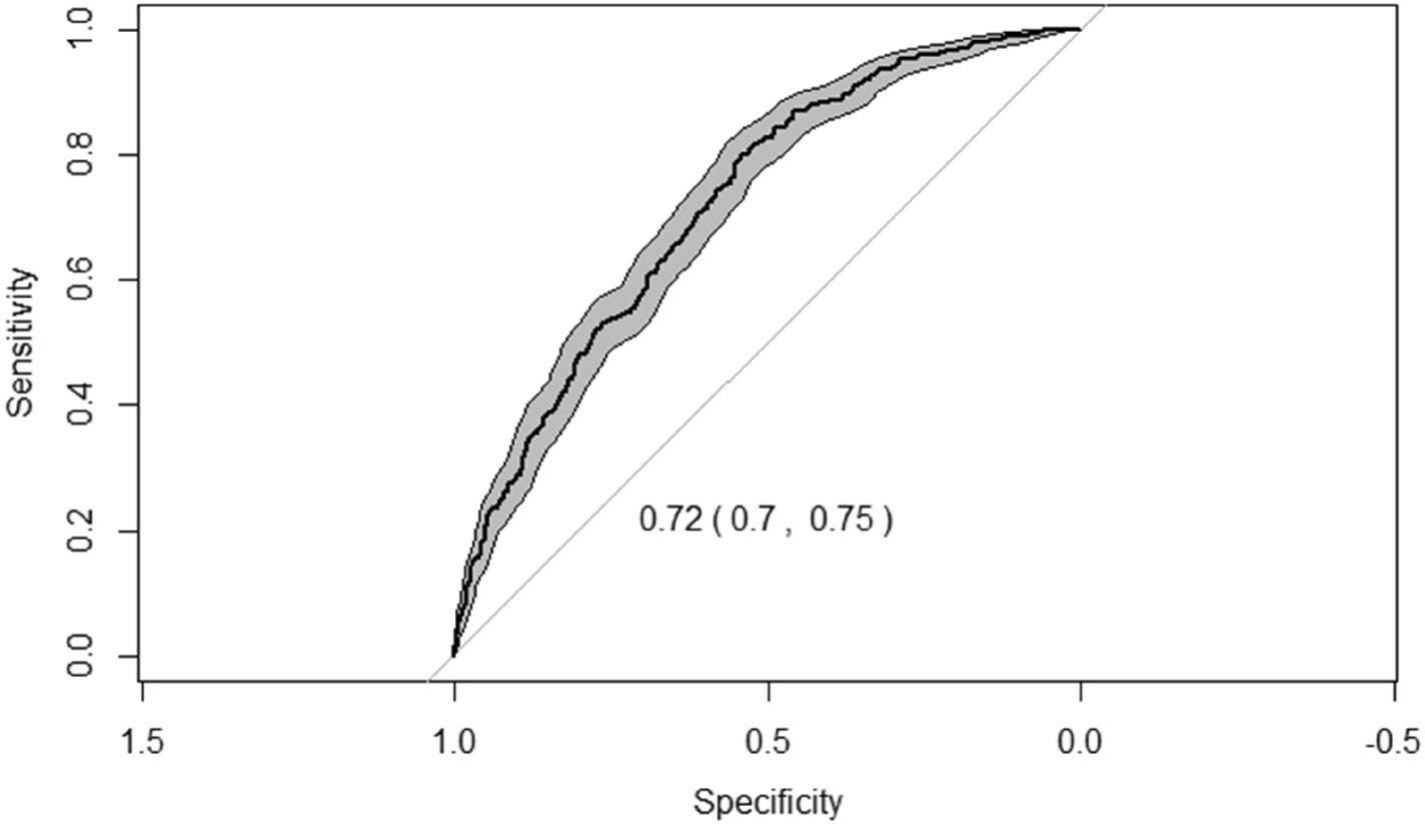
Area under the curve showing the reliability of the CHRONIBERIA index when applied to the global sample.

**Figure 3:**
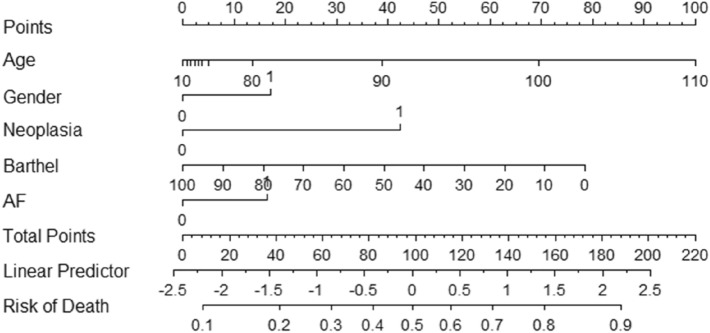
Nomogram, built to facilitate the clinical use of the final index.

At the one-year follow-up, 514 patients (36.6%) had died. The comparison between dead patients and survivors after one year is shown in Table 2. Following the Cox regression analysis, 5 variables were identified as significantly associated to one-year mortality (Fig. 1): age, being male, lower BI punctuation, neoplasia and atrial fibrillation. We created a model with these variables to estimate one-year mortality risk, that led to the CHRONIBERIA index (Table 3). A ROC curve was made in order to determine the reliability of the CHRONIBERIA index when applied to the global sample. An AUC of 0.72 (0.7–0.75) was obtained (Fig. 2) and a nomogram was built to facilitate the clinical use of the final index (Fig. 3).

**Table 2:** Comparison between survivors and dead patients after one-year follow-up, in all patients included in the 4 groups.

Variables	All patients (n = 1406)	Died (n = 514)	Survivors (n = 892)	*P*
Age	79.5 (11.5)	82.8 (9.7)	77.6 (12.1)	** < 0.001**
Gender				
Men	612 (43.5)	223 (43.4)	389 (43.6)	0.935
Women	794 (56.5)	291 (56.6)	503 (56.4)	
Neoplasia				
No	1221 (86.8%)	423 (82.3%)	798 (89.5%)	** < 0.001**
Yes	285 (13.2%)	91 (17.7%)	94 (10.5%)	
BI punctuation	58.2 (34.4)	44.2 (33.5)	66.2 (32.3)	** < 0.001**
PT category				
1	615 (43.7%)	164 (31.9%)	451 (50.6%)	
2	217 (15.4%)	71 (13.8%)	146 (16.4%)	** < 0.001**
3	183 (13%)	71 (13.8%)	112 (12.6%)	
4	100 (7.1%)	47 (9.1%)	53 (5.9%)	
5	291 (20.7%)	161 (31.3%)	130 (14.6%)	
Delirium				
No	1180 (84.4%)	401 (78.6%)	779 (87.7%)	** < 0.001**
Yes	218 (15.6%)	109 (21.4%)	109 (12.3%)	
AF				
No	922 (65.7%)	315 (61.3%)	607 (68.2%)	**0.009**
Yes	482 (34.3%)	199 (38.7%)	283 (31.8%)	
Creatinine, mg/dL	1.35 (0.9)	1.44 (1.1)	1.3 (0.9)	**0.012**

BI: Barthel index, PT: Pfeiffer´s test, AF: Atrial fibrillation.

Significant values are in bold.

**Table 3:** Multivariate logistic regression analysis. Model with the 5 variables to estimate 1-year mortality risk.

	β	SE(β)	OR (95%CI)	*P* value
Intercept	− 0.4898			
Age*, yr	0.5642	0.0984	1.76 (1.45, 2.13)	0.0015
Gender (1, male)	0.4217	0.1301	1.52 (1.18, 1.97)	0.0012
Neoplasia (1, yes)	1.0442	0.1795	2.84 (2.00, 4.04)	< 0.0001
Barthel index	− 0.0193	0.0020	0.42 (0.17, 0.68)	< 0.0001
AF (1, yes)	0.4021	0.1286	1.49 (1.16, 1.92)	0.0018
AUC 0.72	R^2^ 0.19		Brier score 0.20	
AUC corrected 0.72	R^2^ corrected 0.19		Brier score corrected 0.20	

β indicates coefficient; SE, standard error; OR, Odds ratio; CI, 95% confidence interval; *, OR (95%CI) are shown for age interval between 74 and 87 years; AF, Atrial fibrillation; AUC, Area under the ROC curve.

**Figure 4:**
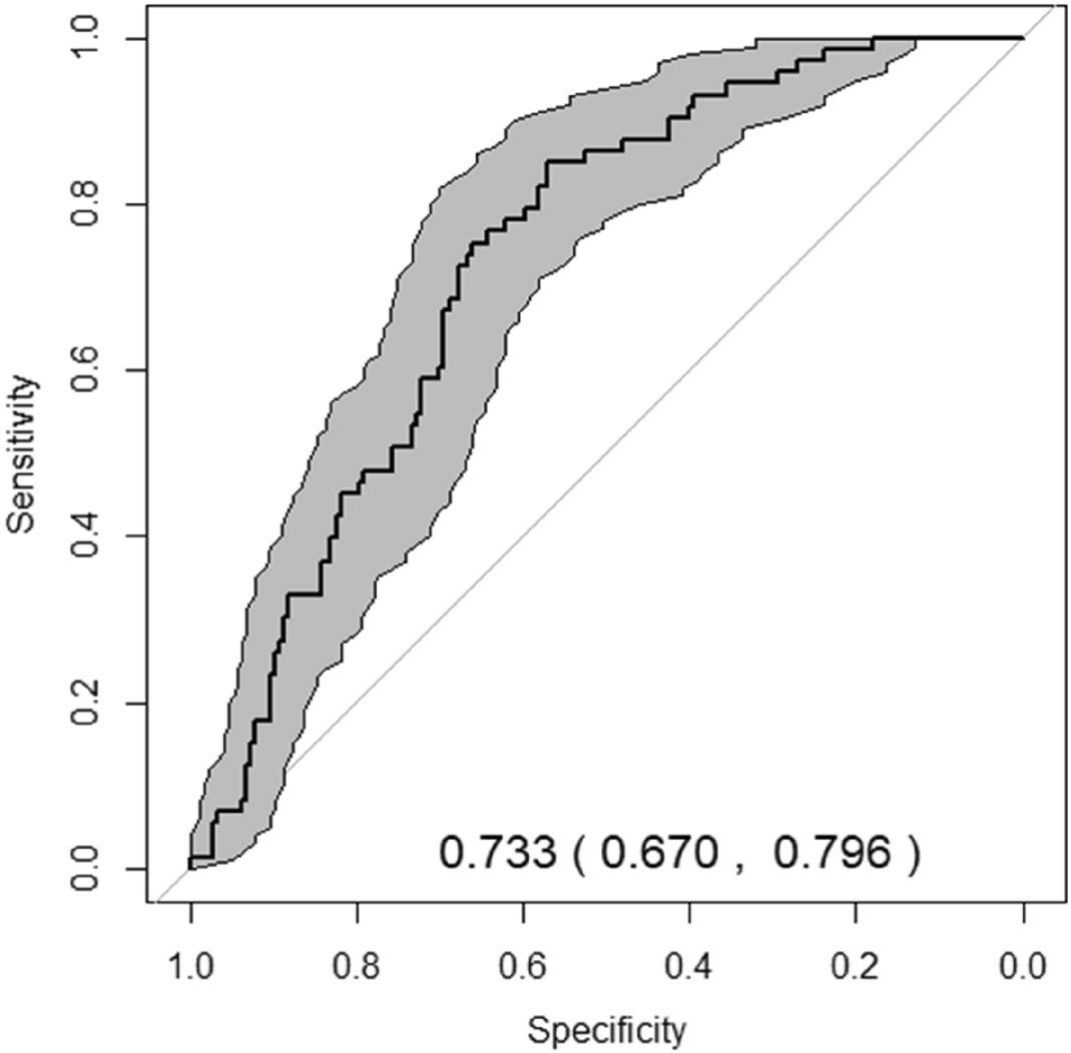
Area under the curve applied to the validation cohort.

Regarding the external validation of the index, it was applied to 250 patients of similar characteristics in the Internal Medicine Department of the Lucus Augusti Hospital (Lugo, Spain). After one year follow-up the ROC curve was nearly equal to that of the original study, with an AUC of 0.73 (0.67–0.79) (Fig. 4).

## Discussion

An optimal approach to chronicity and an its correct care will be critical during the next decade for the healthcare systems of most developed countries^14,19^. In this sense, the availability of powerful tools which can facilitate a correct prognostic stratification of chronic patients could be decisive and lead to an efficient reorganization of healthcare resources^20–22^. To date, we have some disease-specific indexes and scores to evaluate chronic patients, such as those used in chronic pulmonary obstructive disease (COPD)^4^, atrial fibrillation (AF)^23^ or chronic liver disease (CLD)^5^. There are also some scores available focusing on patients with multiple comorbidities but none, to our knowledge, designed specifically for chronic patients, regardless of their age or the number of chronic diseases^8,9,11,24,25^.

In 2016 we described a prognostic index called CRONIGAL, applicable to chronic patients after discharge of Internal Medicine Service^15^. This index included seven variables which were age, neoplasia, delirium, Barthel´s index, Pfeiffer´s test and creatinine. Thus, we focused our analysis in the present study on these variables, with the aim of getting a better correlation with the prognosis of these patients after discharge. The results highlight the importance of atrial fibrillation, regardless its management, as a prognosis factor in these patients with chronic disease and led to the development of a new index by the name of CHRONIBERIA, which is intended to simplify and improve the previous indexes. This index, developed in 4 different cohorts in Spain and Portugal and at different times, achieves good validation in the current cohort of another hospital in our Autonomous Community. This, which could be interpreted as a weakness, we consider to be a strength, in that it gains in generalisability. In short, with samples with different characteristics, a good performance is achieved. The scores for such different samples share similar risks, although AF (data not mentioned in the text) shares a greater weight, as expected in the older population.

The mean age of our series and the higher percentage of females are concordant with previous studies^15,16,24,25^. The grade of physical disability in our series is also similar to that reported in previous studies, showing that even the presence of a single chronic disease can have a huge impact on the patient´s quality of life^11,16^. This fact reinforces the idea that an early extended use of a tool like CHRONIBERIA index in the clinical evaluation of chronic patients could play a key role in the allocation of healthcare resources. In our opinion, this strategy could contribute to reducing the impact of chronic diseases on the quality of life. With regard to cognitive impairment, our series had more patients with severe cognitive impairment than others, but the global percentage of patients with any grade of cognitive impairment was similar^11,16^. The percentage of patients with an active neoplasia was higher than most studies^11,16,26^ but similar to those which included patients with lower ages^24^. In our series only 154 patients (10%) were younger than 65, but we believe that the fact that there was no age restriction better reflects the real influence of cancer in the survival of chronic patients admitted to Internal Medicine departments.

As to mortality, we found that one-year mortality is similar to most previous series that analyzed this variable^11,27,28^. Although these studies were designed for older people or patients with multiple comorbidities, our patients had at least one chronic disease generally associated with several comorbidities. Again, our results highlight the key role of chronicity in the prognosis and survival of patients after discharge. With regard to the variables linked to mortality, age and being male are to be expected, since these are two well-known factors linked to a higher mortality, not only in chronic patients, but also in the general population^11,15,24,27–29^. In the same line, the presence of an active neoplasia is clearly linked to a higher mortality in all circumstances, more so in older people with chronic diseases^11,28^.

The other two variables included in our index deserve a deeper analysis. On the one hand, the presence of functional dependence or physical disability, evaluated through an easy tool like BI can help predict a higher mortality risk, regardless of the presence of cognitive impairment. This fact is, in our opinion, highly relevant and underscores the importance of a functional evaluation in every chronic patient, as other studies had previously shown^30–32^. In the CHRONIBERIA index, the degree of both functional and physical impairment was a key factor, although the cognitive impairment was not. In previous reports cognitive impairment was a determinant variable, linked to functional dependence, but they probably were confounding variables and functional impairment really had a higher relative prognostic value for the survival prognosis of chronic patients^15^. On the other hand, the presence of atrial fibrillation as a marker of mortality risk has been previously reported, mainly in older people with comorbidities^33,34^.

The presence of atrial fibrillation in chronic patients should, in light of our findings, warn physicians of a higher mortality risk. Atrial fibrillation is the most common chronic arrhythmia in older people, with prognostic value demonstrated in heart diseases. In like manner, cardiovascular disease is the main cause of mortality in older people and so, this result should not be strange. In this sense, our group have previously described the prognostic value of atrial fibrillation, regardless of the chronic disease suffered and the type of atrial fibrillation^15^.

We have also subjected the index to an external validation, which clearly confirmed its usefulness and accuracy as a robust tool to be applied in clinical daily practice with chronic patients.

In this paper, we confirm the prognosis importance of atrial fibrillation in chronic patients regardless type of chronic disease and we propose an index of easy application, which could help physicians not only to identify high risk chronic patients, but also to build a healthcare individualized plan, using healthcare and social resources from primary healthcare services. This new index makes an improvement of CRONIGAL index, as includes only 5 variables and the reliability is similar.

The main strength of the CHRONIBERIA index is its easy application in daily clinical practice because consists of 5 variables which are also easy to evaluate. Previous indexes focused on older, multimorbidity or single-disease patients. Consequently, our index makes a difference and provides a new tool to better manage chronicity.

The main limitations of our study could be the differences between the recruitment sites, the differences in recruitment periods and the heterogeneity of the sample as a whole. We believe, however, that the sample size is optimal to validate our results and the enrolment of different types of chronic patients far for being a limitation, demonstrates the usefulness of the CHRONIBERIA index in chronic multimorbidity patients and its potential application in any chronic patient after discharge. In this sense the external validation a few years later, in a similar group, we consider that adds a great value to the CHRONIBERIA index.

In a previous study performed in chronic patients after hospital admission, we have shown that morbidities were not prognostic factors predicting mortality^15^^.^ Therefore, morbidities were not considered as covariates in the models to verify whether atrial fibrillation may be a new prognostic factor for mortality in this group of patients. In the same way, similar studies have shown that frailty (and not comorbidities) is the strongest factor at predicting in-hospital and after discharge mortality in elderly patients^35,36^.

The AUC value could be also criticized, although the simplicity of our index, composed only by 5 variables, makes it, in our opinion, a powerful tool in general medical practice, despite the AUC value. Another potential limitation is the fact that we only used the variables already described in the CRONIGAL index. Reliability might have benefited from the inclusion of additional new variables. On the other hand, as CHRONIBERIA is a prognostic index that can only be applied to chronic patients after discharge, it could not be used in primary healthcare facilities, until the patient had the first decompensation requiring hospital admission.

In conclusion, atrial fibrillation, along with an advanced age, being male, low BI punctuation, or an active neoplasia, could be the critical points to identify a high one-year mortality risk after discharge, in multiple chronic conditions patients Together, these variables constitute the new CHRONIBERIA index.

## Data Availability

The datasets used and/or analyzed during the current study available from the corresponding author on reasonable request.
